# A Text-Book of the Practice of Medicine

**Published:** 1908-06

**Authors:** 


### Reviews of Books.

**A Text-book of the Practice of Medicine.** By HOBART AMORY HARE, M.D., B.Sc. Second Edition. Pp. xv., 1132. London: Henry Kimpton. 1907.

The second edition of Hare's *Practice of Medicine* contains much additional matter, and has undergone extensive revision, so that the book is very considerably enlarged. In carrying out his determination to include nothing that is uncertain and debatable, the author has occasionally been led to omit mention of important views, and even of accepted "symptom-complexes," notably in the case of "myasthenia gravis," which is quite inadequately referred to in the differential diagnosis of "bulbar paralysis." Also in rheumatic fever, as we see it in Europe, at any rate, it is something less than satisfactory when discussing endocarditis and pericarditis to state that "in rarer instances a myocarditis develops." The relative rarity is certainly open to question.

Dr. Hare's advice as to complete disinfection of rooms, clothes and books after influenza seems good, but is rarely acted upon in England. The value of lumbar puncture in doubtful cases of cerebro-spinal fever is undisputed, but the illustration of the operation being performed on a well-developed man sitting upright in complete possession of his senses will not help the novice to operate with success under less favourable circumstances. In a restless, unconscious subject, with some degree of opisthotonos, there are many chances of failure—*experto crede!*

The danger of antipyretic drugs in croupous pneumonia is rightly insisted upon, and marks the timely revolt against a treatment that has for long enough pretended to a scientific justification. Venesection, on the other hand, meets with the just praise which but recently was denied to it. In fact, the treatment throughout the book is clearly detailed, temperately and accurately appraised, and the indications made thoroughly plain. There are very few text-books which deal with the subject more satisfactorily.

On the American Continent the opportunities for studying tropical diseases are perhaps more numerous and more accessible than in any other part of the world, and it is not surprising to find that particular attention has been paid to this branch of medicine; it is at the same time, gratifying to our insular pride to find the work of the English pioneers—Manson, Ross, Bruce, Leishman and Wright, with many others—handsomely acknowledged.

Dr. Hare's chapters on tropical diseases old and new give briefly and intelligibly a complete account of the most recent work that has been done throughout the world, and in these days of rapid travel even the most stay-at-home practitioner may find himself confronted with the strange maladies of other climes.

We cannot refrain from saying of the literary style that in America, thanks, no doubt, to the influence of Holmes, Emerson, Prescott and Motley, our common language seems to fare better at the hands of scientific writers than it is wont to do in twentieth-century England: this book is readable.

